# Age, Motivation, and Emotion Regulation Skills Predict Treatment Outcome in an Internet-Based Self-Help Intervention for COVID-19 Related Psychological Distress

**DOI:** 10.3389/fpubh.2022.835356

**Published:** 2022-06-09

**Authors:** Noemi Anja Brog, Julia Katharina Hegy, Thomas Berger, Hansjörg Znoj

**Affiliations:** ^1^Department of Health Psychology and Behavioral Medicine, University of Bern, Bern, Switzerland; ^2^Department of Clinical Psychology and Psychotherapy, University of Bern, Bern, Switzerland

**Keywords:** COVID-19, internet-based self-help, depressive symptoms (DSs), psychological distress, resilience

## Abstract

**Introduction:**

First evidence suggests that internet-based self-help interventions effectively reduce COVID-19 related psychological distress. However, it is yet unclear which participant characteristics are associated with better treatment outcomes. Therefore, we conducted secondary analyses on data from a randomized controlled trial investigating the efficacy of a 3-week internet-based self-help intervention for COVID-19 related psychological distress. In this exploratory analysis, we examined several predictors ranging from sociodemographic variables to psychological distress, resource-related, and treatment-related variables. This includes, for example, age, motivation, and emotion regulation skills. Treatment outcomes were defined as post-treatment depressive symptoms and post-treatment resilience.

**Methods:**

In a total of 107 participants with at least mild depressive symptoms, possible predictor variables and treatment outcomes were assessed using self-report measures. For example, emotion regulation skills were assessed by the Self-report measure for the assessment of emotion regulation skills. In a first step, we performed a separate linear regression analysis for each potential predictor. In a second step, predictors meeting a significant threshold of *p* < 0.05 were entered in linear multiple regression models. Baseline scores of the respective outcome measure were controlled for.

**Results:**

The mean age of the participants was 40.36 years (SD = 14.59, range = 18–81 years) with the majority being female (*n* = 87, 81.3%). Younger age predicted lower post-treatment depressive symptoms. Additionally, higher motivation to use the intervention and better pre-treatment emotion regulation skills predicted higher post-treatment resilience.

**Conclusion:**

The current study provides preliminary evidence regarding the relationship between participant characteristics and treatment outcome in internet-based self-help interventions for COVID-19 related distress. Our results suggest that under the circumstances surrounding COVID-19 such interventions might be particularly beneficial for young adults regarding depressive symptoms. Moreover, focusing on participants' existing strengths might be a promising approach to promote resilience through internet-based self-help interventions. However, since this was an exploratory analysis in an uncontrolled setting, further studies are needed to draw firm conclusions about the relationship of participant characteristics and treatment outcome in internet-based self-help interventions for COVID-19 related psychological distress.

## Introduction

In March 2020, the World Health Organization (WHO) declared the COVID-19 (acute respiratory syndrome coronavirus 2; SARS-CoV-2) outbreak a pandemic (1). At the onset and during the COVID-19 pandemic, studies indicated a deterioration of mental health in the general population (2–6). In particular, evidence for an increase in depression and anxiety symptoms was found (7). For example, in a study in the USA, a tripling of the prevalence of depression symptoms in the general population during the COVID-19 pandemic was reported (8). However, some studies reported that the COVID-19 pandemic and associated restrictions had no impact or even a positive impact on the wellbeing of the general population (9, 10). For example, Aghababa et al. (10) found stable activity patterns among team sport athletes and Albrecht et al. (9) report positive effects of homeschooling on adolescent sleep schedules. Moreover, some studies indicated that the initial increase in psychological distress in the general population decreased over the course of the COVID-19 pandemic (11–13). These findings suggest that on average, the general population might be resilient. Resilience can be defined as the maintenance of stable mental health or recovery from initial psychological distress in the face of major life stressors (14). Nonetheless, a substantial minority of the general population shows heightened psychological distress (15, 16). Accordingly, mental health interventions mitigating this psychological distress are needed.

A promising approach is the use of internet-based self-help interventions since they do not require direct on-site contact and are easily scalable (17–19). Studies indicate that internet-based self-help interventions are an effective treatment option for various psychological problems, including depressive symptoms (20, 21). So far, few studies have investigated the efficacy of internet-based self-help interventions for COVID-19 related psychological distress in the general population. However, first results suggest that internet-based self-help interventions are efficacious in reducing COVID-19 related worry and associated symptoms (22), symptoms of depression, anxiety, and stress (23, 24), as well as in promoting resilience and emotion regulation skills (25). Nonetheless, in one study, there was no significant reduction of depressive symptoms (25). Given that there is still comparatively little research on internet-based self-help interventions for COVID-19 related psychological distress and that it shows mixed results, it is important to find out who might benefit from internet-based self-help for COVID-19 related psychological distress.

In the context of the COVID-19 pandemic, understanding the relationship between participant characteristics and treatment outcome is of particular interest since some studies point toward the need for tailoring interventions for specific risk populations (5, 7). Identifying predictors of treatment outcomes in internet-based self-help for COVID-19 related psychological distress would allow interventions to be tailored to specific needs and thus improve intervention efficacy. Accordingly, knowledge of the predictors of treatment outcomes would inform how interventions could be improved for specific use in target populations or adapted for other target populations. For example, if age predicts treatment outcomes, interventions could be tailored and improved for specific age groups or adapted for those not yet reached. So far, potential risk factors for heightened psychological distress due to the COVID-19 pandemic include for example: pre-existing mental health problems (26–28), pre-existing physical health problems (27), younger age (29–32), identifying as non-binary (27), female gender (27–30), and difficulties in emotion regulation (33, 34).

However, no study to date has investigated predictors of treatment outcome in internet-based self-help interventions for COVID-19 related psychological distress. Therefore, to improve the understanding of the relationship between participant characteristics and treatment outcome in internet-based self-help for COVID-19 related psychological distress, we explored predictors of treatment outcome in an internet-based self-help intervention for COVID-19 related psychological distress called ROCO (25, 35). ROCO is an acronym for *Resilience and Optimism during COVID-19*. The 3-week ROCO intervention included guidance on demand and the participants had the possibility to contact a psychologist *via* a chat function. The efficacy of the ROCO intervention was evaluated in a randomized controlled trial, from which the data used in this study are drawn (25). The primary target of the ROCO intervention was a reduction of depressive symptoms. However, a considerable part of the intervention was also aimed at promoting resilience (35). Therefore, in the present study, we defined treatment outcomes as post-treatment depressive symptoms and post-treatment resilience. In the RCT, the 3-week ROCO intervention did not significantly reduce primary depressive symptoms and secondary outcomes such as anxiety and stress symptoms. Moreover, the intervention had no beneficial effects on secondary outcomes such as quality of life, optimism, embitterment, loneliness, and self-efficacy. However, the intervention led to a significant increase in emotion regulation skills and resilience (small-to-medium effect sizes).

The aim of this exploratory analysis is to investigate possible predictors of treatment outcome in an internet-based self-help intervention for COVID-19 related psychological distress. Specifically, we aimed to explore, who improves more during treatment. Based on the above mentioned previous research on possible risk factors for COVID-19 related psychological distress, we decided to explore sociodemographic variables (age, gender, and level of education), psychological distress variables (ever having received a psychiatric diagnosis, previous or current psychotherapy, current medication, anxiety, stress, embitterment, loneliness, and mental and physical health quality), and resource-related variables (emotion regulation skills, optimism, and self-efficacy) as possible predictors. Moreover, we explored if treatment-related variables (motivation to use the self-help intervention, number of completed modules) predict treatment outcome.

## Materials and Methods

### Study Design

The data used in the current study were obtained in a parallel-group randomized controlled trial (RCT) investigating the efficacy of a short internet-based self-help intervention for COVID-19 related psychological distress called ROCO. The protocol of the study was approved by the Ethics Committee of the Canton of Bern, and the trial was registered on ClinicalTrials.gov (NCT04380909).

In the RCT, an immediate treatment group was compared to a waiting control group, with both groups receiving care as usual [CAU; (25, 35)]. Participants were randomized in a 1:1 ratio to either the immediate treatment group or the waiting control group. Participants in the immediate treatment group received direct access to the 3-week internet-based ROCO intervention, whereas participants in the waiting control group had a waiting period of 3 weeks and then received access to the ROCO intervention (i.e., delayed treatment). Three weeks after randomization, all participants had to fill out a second assessment (post-treatment for the immediate treatment group; pre-treatment for the waiting control group). All participants had to complete a third assessment 6 weeks after the randomization (follow-up for the immediate treatment group; post-treatment for the waiting control group). In the RCT, analyses were conducted according to an intention-to-treat principle (25).

For the present secondary analysis, data from both groups were combined, using the data of the respective treatment phase (immediate or delayed). The investigated predictors of post-treatment outcomes (depressive symptoms and resilience, respectively) were assessed before the respective treatment phase (i.e., for the immediate treatment group at baseline and for the waiting control group after the waiting period). Sociodemographic variables as well as information on previous or current psychological treatments (ever received a psychiatric diagnosis, prior experience with psychotherapy, current psychotherapy, or medication intake) were collected for both groups at baseline.

### Participants

We recruited German-speaking participants between April 2020 and February 2021, primarily through newspaper articles, internet forums on mental health, and advertisements on the internet. Interested participants registered on our study homepage and subsequently received the detailed study information. After providing informed consent, participants completed the online baseline assessment, consisting of questions concerning socio-demographic variables, previous or current psychological treatment, and various self-report questionnaires.

The following inclusion and exclusion criteria were evaluated based on this baseline assessment: participants had to be at least 18 years of age, have access to the internet, show sufficient knowledge of the German language, provide an emergency address for the case of an acute crisis, and reach a minimum of 4 points on the Patient Health Questionnaire [PHQ; (36)], which is interpreted as the presence of mild depressive symptoms. Participants were excluded if they reached a cut-off value of 8 points on the Suicide Behavior Questionnaire [SBQ-R; (37)], which would indicate the presence of suicidal tendencies. Furthermore, participants reporting a known psychotic or bipolar disorder diagnosis were also excluded. A total of 107 participants met all the inclusion criteria and none of the exclusion criteria, thus constituting the current study sample.

### Measures

All assessments were administered online and consisted of self-report questionnaires. We used the German versions of the self-report questionnaires.

#### Outcome Measures

Depressive symptoms, the primary treatment target of the internet-based intervention, were measured with the PHQ-9 (36). The PHQ-9 is used to assess the severity of depressive symptoms. For this purpose, nine items are scored on a scale from 0 = not at all to 5 = nearly every day. The items are introduced as follows: “Over the last 2 weeks, how often have you been bothered by any of the following problems?” Examples of items are: “Little interest or pleasure in doing things” and “Feeling down, depressed, or hopeless.” The nine items correspond to the nine DSM-IV criteria for depression. From the nine items, a score is built: a score of 5 corresponds to mild depression, a score of 10 to moderate depression, a score of 15 to moderately severe depression, and a score of 20 to severe depression (38). In the present sample, the PHQ-9 had a satisfactory internal consistency (Cronbach's α = 0.72 at pre-treatment and Cronbach's α = 0.74 at post-treatment).

A secondary treatment target of the internet-based intervention was to promote resilience. Resilience was measured with the Connor-Davidson Resilience Scale [CD-RISC; (39)]. In the present study, the 10-item version of the CD-RISC was used. Examples of items are: “I am able to adapt to change” and “I can handle unpleasant feelings.” The 10 items are answered on a scale from 0 = not true at all to 4 = true nearly all of the time. Higher scores correspond to more resilience. In the present sample, the CD-RISC showed good internal consistency (Cronbach's α = 0.88 at pre-treatment and Cronbach's α = 0.90 at post-treatment).

#### Predictors

We grouped a total of 18 possible predictor variables into four groups: sociodemographic, psychological distress, resource-related, and treatment-related variables.

Sociodemographic Variables. We assessed *age, gender*, and *level of education*.

##### Psychological Distress Variables

At baseline, we assessed whether participants had *ever received a psychiatric diagnosis*, had *previously been in psychotherapy*, were *currently in psychotherapy*, and were *currently taking medication for mental health problems*. These variables were chosen as measures of pre-existing mental health problems and current psychological treatment needs, indicative of psychological burden (13, 26).

At pre-treatment, we assessed several variables using self-report questionnaires. *Anxiety* and *stress* were measured by the corresponding subscales of the DASS-21 (40). Each subscale consists of seven items, which are answered on a scale from 0 = did not apply to me at all to 3 = applied to me very much or most of the time. Examples of items are: “I found it hard to wind down,” “I felt I was close to panic,” and “I was unable to become enthusiastic about anything.” On the anxiety subscale, a score of 4 represents mild anxiety, a score of 6 moderate anxiety, a score of 8 severe anxiety, and a score of 10 extremely severe anxiety. On the stress subscale, a score of 8 represents mild stress, a score of 10 moderate stress, a score of 13 severe stress, and a score of 17 extremely severe (41). In the present sample, the internal consistency at pre-treatment was close to satisfactory for the anxiety subscale (Cronbach's α = 0.68) and good for the stress subscale (Cronbach's α = 0.81).

*Mental health quality* and *physical health quality* were assessed as measures of general health-related quality of life with the respective scales of the 12-Item Short-Form Health Survey (27, 42). An example of an item is: In general, would you say your health is: Excellent, Very Good, Good, Fair, or Poor. Higher scores on the respective subscale indicate better mental health quality, respectively physical health quality. The SF-12 has a good test-retest reliability (43).

*Embitterment* was measured with the 6-item version of the Bern Embitterment Inventory (44). Embitterment can be defined as the feeling of being disadvantaged by others and fate and might be a mental health reaction to the COVID-19 pandemic (45–47). Examples of items are: “It fills me with bitterness when I think of the goals that have not been achieved” and “I sometimes think that all people are bad and corrupt.” Items are scored on a scale from 0 = I do not agree to 4 = I agree, with higher scores representing more embitterment (48). In the present sample, the internal consistency of the BEI at pre-treatment was good (Cronbach's α = 0.80).

*Loneliness* was assessed using the 9-item version of the UCLA Loneliness Scale [ULS; (49)] since several studies postulated a link between loneliness and mental health problems and the COVID-19 pandemic has been reported to increase loneliness (50, 51). Examples of items are: “How often do you feel that there are people you can talk to?” and “How often do you feel isolated from others?.” The items are answered on a scale from 1 = never to 4 = often, with higher scores indicating more loneliness. In the present sample, the internal consistency of the ULS at pre-treatment was good (Cronbach's α = 0.85).

##### Resource-Related Variables

We assessed several resource-related variables using self-report questionnaires at pre-treatment. *Self-efficacy* Was assessed using the General Self-Efficacy Scale [GSE; (52)]. The 10 items Are scored on a scale From 1 = Not at all true to 4 = exactly true, With higher scores indicating more self-efficacy (52). Examples of items Are: “It Is easy for me to stick to my aims and accomplish my goals” and “I can solve most problems if I invest the necessary effort.” In the present sample, the internal consistency of the GSE at pre-treatment was good (Cronbach's α = 0.89).

*Optimism* was assessed with the Life Orientation Test Revised [LOT-R; (53)]. The total score of the 10-item LOT-R is built from six items, since four items are filler items. A higher score indicates more optimism. Examples of items are: “In uncertain times, I usually expect the best” and “I rarely count on good things happening to me.” The items are answered on a scale from 0 = strongly disagree to 4 = strongly agree (53). In the present sample, the internal consistency of the LOT-R at pre-treatment was good (Cronbach's α = 0.84).

*Emotion regulation skills* were assessed with the Self-report measure for the assessment of emotion regulation skills [SEK-27; (54)]. The 27 items of the SEK-27 are answered on a scale from 0 = never to 4 = (almost) always, with higher scores corresponding to better emotion regulation skills (54). The items are introduced as follows: “Dealing with emotions: Last week....” Examples of items then are: “I understood my emotional reactions,” “I was OK with my feelings, even if they were negative,” and “I supported myself in emotional distressing situations.” In the present sample, the internal consistency for the SEK-27 at pre-treatment was excellent (Cronbach's α = 0.93).

##### Treatment-Related Variables

*Motivation* to use the internet-based intervention was assessed at baseline with one item (Please indicate your motivation to use the ROCO program in general). Participants could rate their motivation with a regulator From 0 = no motivation at all to 100 = greatest possible motivation.

The *number of completed modules* was measured after the treatment. It could range from 0 (no module completed) to 6 (all modules completed).

### Description of Intervention

The internet-based self-help intervention ROCO was aimed at persons experiencing COVID-19 related psychological distress. The 3-week intervention consisted of six thematic modules, an introduction, and a conclusion. Additionally, the intervention comprised a page with information on what to do in an acute crisis, including a list of emergency contacts, a page with an overview of the weekly exercises, and a page with a symptom-tracking questionnaire, allowing participants to track their self-reported symptoms. The six thematic modules were based on cognitive-behavioral therapy and included texts, videos, graphics, and exercises. Each thematic module had a specific focus: in module 1, psychoeducation about COVID-19 related psychological distress was given. In module 2, participants learned about emotions and emotion regulation. In module 3, the identification and restructuring of thought patterns were addressed. In module 4, participants acquired knowledge about several possibilities to promote resilience. In module 5, relaxation techniques and sleep hygiene were discussed. Finally, in module 6, the topics of self-care and personal growth were approached. For a more detailed description of the intervention, see the study protocol of the ROCO RCT (35). Participants were advised to work through two modules per week. However, the participants could decide for themselves which modules they wanted to work on and in which order. A module took about 40 to 80 mins to complete. Since the internet-based self-help program offered guidance on demand, the participants had the possibility to contact a psychologist *via* a chat function. The psychologist answered within three working days. Otherwise, there was no scheduled contact.

### Statistical Analysis

All analyses were conducted using IBM SPSS Statistics (version 25). Independent samples *t*-tests and χ^2^-tests (for nominal data) were performed to examine group differences at baseline and pre-treatment. In a first step, potential predictors were identified using simple linear regression analyses. For each potential predictor a separate linear regression analysis was performed as follows: the potential predictor (e.g., age) was entered as predictor, the post-treatment score of the outcome (depressive symptoms or resilience) was entered as dependent variable, and the pre-treatment score of the respective outcome (e.g., depressive symptoms) was defined as covariate. We predetermined, that predictors had to reach a *p*-value below 0.05 to be included in the subsequent multiple regression analyses. In a second step, a multiple regression analysis was performed for each outcome with the predictors identified in step 1 entered as predictors and the pre-treatment score of the respective outcome entered as covariate. All tests were two-sided. This approach of stepwise selection of predictors has been criticized (e.g., for leading to bias in predictor effects or variability of selected predictors) and modern prediction methods have been recommended (55, 56). Accordingly, stepwise regression procedures are unfavorable for explanatory purposes. However, since stepwise regression procedures might be of value for exploratory data analysis (57), we refrained from using modern prediction methods.

To account for possible group effects, we additionally tested whether group (immediate vs. delayed treatment) was a significant predictor for the outcome while using the pre-treatment values of the respective outcome as covariate. If the group was a significant predictor (*p* < 0.050), it was added as covariate in the multiple regression analysis of the respective outcome. We did not replace missing data in the predictor variables. Hence only participants with complete data sets were considered for the respective outcomes (completers analysis).

The assumptions for performing multiple linear regressions were checked in advance (58). Our sample size was *N* = 107 > 100. However, the number of predictors times ten (18 × 10 = 180) was greater than our sample size, possibly leading to overfitting of regression models. Yet even two observations per parameter have been found to not bias the estimate in linear regression analysis (59). The variances inflation factors (VIF) were between 1 and 2.732 whereas a VIF <1 and VIF > 10 indicates multicollinearity and the predictors explained the dependent variables (*Rs* = 0.545–0.818, *R*^2^ *s* = 0.297–0.669). We did not calculate Durbin-Watson coefficients because our dependent variable (respective treatment outcome) was also in the model as an independent variable (pre-treatment score of the respective outcome) with a time lag and thus the application of the Durbin-Watson statistic is not warranted (60). Moreover, we inspected the distribution of the residuals (homoscedasticity and normal distribution) and checked for outliers. We identified one outlier based on the studentized residuals, however Cook's distances indicated that the case was not influential for our models (61). Therefore, we did not remove this outlier from the data (62).

## Results

### Sample Characteristics

The total sample consisted of 107 German-speaking participants. On average, they were 40.36 years old (*SD* = 14.59, range = 18–81 years) and the majority were female (*n* = 87, 81.3%), had a university degree (*n* = 64, 59.8%) and previous experience with psychological treatment (*n* = 68, 63.6 %). Overall, 28 participants (26.2%) were in concurrent psychological treatment and 24 participants (22.4%) were taking medication for psychological problems at baseline. The participants showed, on average, moderate depressive symptoms (*M* = 10.37, *SD* = 4.18) and mild anxiety and stress symptoms (*M* = 4.33, *SD* = 3.26; *M* = 8.80, *SD* = 4.10) at pre-treatment. Approximately one third of the participants (*n* = 36, 33.6 %) reported having received a psychiatric diagnosis at some point in their lives. Baseline or pre-treatment scores of the predictor variables and outcome measures are displayed in Table 1. There was a significant group difference in terms of education (${\text{χ}}_{\left(1\right)}^{2}$ = 5.055, *p* = 0.025), indicating that participants in the immediate treatment group had a lower average level of education. Moreover, participants in the delayed treatment group completed significantly fewer modules of the intervention than participants in the immediate intervention group (*t*_(104.1)_ = 2.719, *p* = 0.009). Additionally, the delayed treatment group showed markedly lower pre-treatment depression scores compared to the immediate treatment group [immediate treatment group *M* (SD) = 11.13 (4.36) vs. delayed treatment group *M* (SD) = 9.60 (3.89)]. However, the group difference was not significant (*t*_(102.1)_ = 1.908, *p* = 0.055).

**Table 1:** Predictors and outcome measures at baseline or pre-treatment, overall and divided by group.

	**Total *N* = 107**	**Immediate treatment group *n* = 53**	**Delayed treatment group *n* = 54**	**Statistic**
**Socio-demographic variables**				
Age, *M (SD)*	40.36 (14.59)	40.68 (15.55)	40.04 (13.73)	*t*_(105)_ = 0.227, *p* = 0.819^*b*^
Female, *n* (%)	87 (81.3)	46 (86.8)	41 (75.9)	$X_{\left(1\right)}^{2}$ = 2.078, *p* = 0.149
University, *n* (%)	64 (59.8)	26 (49.1)	38 (70.4)	$X_{\left(1\right)}^{2}$ = 5.055, *p* = 0.025
**Psychological distress variables**				
Psychiatric diagnosis, *n (%)*	36 (33.6)	21 (39.6)	15 (27.8)	$X_{\left(1\right)}^{2}$ = 1.681, *p* = 0.195
Psychological treatment				
Previous, *n* (%) Current, *n* (%)	68 (63.6) 28 (26.2)	38 (71.7) 14 (26.4)	30 (55.6) 14 (25.9)	$X_{\left(1\right)}^{2}$ = 3.009, *p* = 0.083 $X_{\left(1\right)}^{2}$ = 0.003, *p* = 0.954
Current medication, *n* (%)	24 (22.4) n^*a*^ = 105	14 (26.4) *n* = 52	10 (18.5) *n* = 53	$X_{\left(1\right)}^{2}$ = 0.966, *p* = 0.326
Anxiety (DASS-21), *M (SD)*	4.33 (3.26) *n* = 105	4.43 (3.51)	4.23 (3.01) *n* = 52	*t*_(101.3)_ = 0.319, *p* = 0.741^*b*^
Stress (DASS-21), *M (SD)*	8.80 (4.10) *n* = 105	9.42 (4.03)	8.17 (4.12) *n* = 52	*t*_(103)_ = 1.562, *p* = 0.119^*b*^
Embitterment (BEI), *M (SD)*	9.12 (5.04) *n* = 103	8.75 (4.88)	9.50 (5.22) *n* = 50	*t*_(101)_ = −0.749, *p* = 0.440^*b*^
Loneliness (ULS), *M (SD)*	20.77 (4.46) *n* = 105	21.26 (4.82)	20.27 (4.04) *n* = 52	*t*_(100.6)_ = 1.147, *p* = 0.261^*b*^
Mental health quality (SF-12), *M, (SD)*	31.66 (9.12) *n* = 105	31.10 (9.10)	32.23 (9.20) *n* = 52	*t*_(103)_ = −0.636, *p* = 0.528^*b*^
Physical health quality (SF-12), *M (SD)*	53.65 (7.68) *n* = 105	53.43 (8.79)	53.87 (6.43) *n* = 52	*t*_(95.3)_ = −0.292, *p* = 0.779^*b*^
**Resources**				
Optimism (LOT-R), *M (SD)*	14.33 (4.89) *n* = 103	14.43 (5.04)	14.22 (4.73) *n* = 50	*t*_(101)_ = 0.222, *p* = 0.820^*b*^
Self-efficacy (GSE), *M (SD)*	26.29 (4.47) *n* = 104	25.91 (4.47)	26.69 (4.47) *n* = 51	*t*_(102)_ = −0.890, *p* = 0.369^*b*^
Emotion regulation skills (SEK-27), *M (SD)*	62.70 (15.97) *n* = 103	62.64 (15.45)	62.76 (16.65) *n* = 50	*t*_(101)_ = −0.037, *p* = 0.976^*b*^
**Treatment-related variables**				
Number of completed modules, *M (SD)*	3.51 (2.47)	4.15 (2.27)	2.89 (2.53)	*t*_(104.1)_ = 2.719, *p* = 0.009^*b*^
Motivation, *M (SD)*	84.26 (14.14)	83.09 (17.20)	85.41 (10.35)	*t*_(85.0)_ = −0.841, *p* = 0.417^*b*^
**Outcome measures**				
Depressive symptoms (PHQ-9), *M (SD)*	10.37 (4.18) *n* = 105	11.13 (4.36)	9.60 (3.89) *n* = 52	*t*_(102.1)_ = 1.908, *p* = 0.055^*b*^
Resilience (CD-RISC), *M (SD)*	22.47 (6.68) *n* = 103	21.87 (6.62)	23.10 (6.75) *n* = 50	*t*_(101)_ = −0.935, *p* = 0.359^*b*^

*M, Mean; SD, standard deviation; DASS-21, Depression Anxiety Stress Scale; BEI, Bern Embitterment Inventory; ULS, UCLA Loneliness Scale; SF-12, Short-Form Health Survey; LOT-R, Life Orientation Test Revised; GSE, General Self-Efficacy Scale; SEK-27, Self-report Measure to Measure Emotion Regulation Skills; PHQ-9, Patient Health Questionnaire; CD-RISC, Connor-Davidson Resilience Scale*.

a *N's range from 103 to 107 due to occasional missing data. If n is not reported, it equals the number in the column header*.

b *Bootstrap 1,000 samples*.

### Identifying Predictors of Post-treatment Depressive Symptoms and Resilience

In a first step, variables predicting post-treatment depressive symptoms and resilience were identified using simple linear regressions. We controlled for pre-treatment scores of the corresponding outcome measures (depressive symptoms or resilience). The results of the single predictor analysis are displayed in Table 2. In a second step, the variables that met the previously defined threshold of a *p*-value below 0.05 were included in a multiple regression model (see Tables 3, 4). All models used centered predictor variables (grand mean-centered) to anticipate possible multicollinearity. Since the variable group (immediate vs. delayed treatment) was a significant covariate for resilience (Δ*R*^2^ = 0.034, β = −0.184, *p* = 0.013), it was entered in the respective multiple regression.

**Table 2:** Single-predictor linear regression analysis with post-treatment depressive symptoms respectively post-treatment resilience as dependent variable controlling for pre-treatment depressive symptoms, respectively pre-treatment resilience.

	**Depressive symptoms (PHQ-9)**			**Resilience (CD-RSIC)**		
**Predictors**	Δ***R^**2**^***	***β***	***p***	Δ***R^**2**^***	***β***	***p***
**Socio-demographic variables**						
Age	0.066	0.259	0.006	0.004	0.066	0.382
Female Gender	0.019	−0.138	0.145	0.009	0.096	0.207
University	0.022	0.148	0.119	0.002	0.043	0.571
**Psychological distress variables**						
Anxiety (DASS-21)	0.044	0.246	0.026	0.000	0.005	0.950
Stress (DASS-21)	0.036	0.238	0.044	0.000	0.018	0.821
Embitterment (BEI)	0.001	0.030	0.767	0.011	0.113	0.158
Loneliness (ULS)	0.006	0.083	0.422	0.009	0.102	0.200
Mental health quality (SF-12)	0.000	0.012	0.925	0.000	0.021	0.794
Physical health quality (SF-12)	0.031	−0.178	0.063	0.001	−0.026	0.737
Psychiatric diagnosis	0.056	−0.237	0.011	0.000	0.002	0.982
Previous psychotherapy	0.069	−0.263	0.005	0.005	0.070	0.353
Current psychotherapy	0.063	−0.251	0.007	0.007	0.086	0.254
Current medication	0.005	−0.070	0.475	0.002	−0.045	0.573
**Resources**						
Self-efficacy (GSE)	0.011	−0.114	0.276	0.007	0.139	0.267
Optimism (LOT-R)	0.010	−0.103	0.302	0.000	0.008	0.934
Emotion regulation skills (SEK-27)	0.008	−0.103	0.349	0.024	0.189	0.037
**Treatment-related variables**						
Number of completed modules	0.026	−0.162	0.086	0.003	0.054	0.475
Motivation	0.020	0.141	0.135	0.027	0.163	0.030

*Block one: pre-treatment depressive symptoms (R^2^ = 0.297, β = 0.545, p < 0.001), respectively pre-treatment resilience (R^2^ = 0.580, β = 0.762, p < 0.001). Block two: predictor variables. PHQ-9, Patient Health Questionnaire; CD-RISC, Connor-Davidson Resilience Scale; DASS-21, Depression Anxiety Stress Scale; BEI, Bern Embitterment Inventory; ULS, UCLA Loneliness Scale; SF-12, Short-Form Health Survey; GSE, General Self-Efficacy Scale; LOT-R, Life Orientation Test Revised; SEK-27, Self-report Measure to measure emotion regulation skills*.

**Table 3:** Predictors of the post-treatment depressive symptoms (multiple regression).

	**Depressive symptoms**		
**Predictors**	***b (SE)***	***t***	***p***
Pre-treatment depressive symptoms	0.299 (0.094)	3.193	0.002
Age	0.043 (0.020)	2.184	0.032
Anxiety (DASS-21)	0.179 (0.114)	1.565	0.122
Stress (DASS-21)	0.188 (0.096)	1.971	0.053
Psychiatric diagnosis	−0.763 (0.704)	−1.084	0.282
Previous psychotherapy	−1.313 (0.726)	−1.808	0.075
Current psychotherapy	−0.864 (0.768)	−1.125	0.264

*The model was significant (F_(7,73)_ = 10.715, p < 0.001), adjusted R^2^ = 0.459; the model includes an intercept (b = 10.304, SE = 0.62, t = 16.650, p < 0.001); predictors were selected based on single-predictor regressions (Table 2); predictors were grand-mean centered to avoid multicollinearity*.

**Table 4:** Predictors of the post-treatment resilience (multiple regression).

	**Resilience**		
**Predictors**	***b (SE)***	***t***	***p***
Pre-treatment resilience	0.691 (0.086)	8.007	<0.001
Group (immediate vs. delayed treatment)	−2.465 (0.917)	−2.687	0.009
Emotion regulation skills (SEK-27)	0.072 (0.036)	2.023	0.047
Motivation	0.092 (0.032)	2.851	0.006

*The model was significant (F_(4,71)_ = 35.858, p < 0.001), adjusted R^2^ = 0.650; the model includes an intercept (b = 23.790, SE = 0.61, t = 38.857, p < 0.001); predictors were selected based on single-predictor regressions (Table 2); predictors were grand-mean centered to avoid multicollinearity*.

### Predictors of Post-treatment Depressive Symptoms in Multiple Regression

Within the first multiple linear regression, we examined predictors for post-treatment depressive symptoms (see Table 3). The age of the participants at baseline was a significant predictor of post-treatment depressive symptoms [*b* (SE) = 0.043 (0.020), *p* = 0.032]. The older the participants were, the higher their depressive symptoms were post-treatment.

### Predictors of Post-treatment Resilience in Multiple Regression

Table 4 displays the results of the second multiple linear regression, in which post-treatment resilience was the outcome. Both motivation at baseline [*b* (SE) = 0.092 (0.032), *p* = 0.006] and pre-treatment emotion regulation skills [*b* (SE) = 0.072 (0.036), *p* = 0.047] predicted post-treatment resilience. The higher the motivation of the participants to use the intervention was, the higher their resilience was post-treatment. Likewise, the better the emotion regulation skills of the participants were pre-treatment, the higher their resilience was post-treatment.

## Discussion

In the present study, we aimed to identify predictors of treatment outcome in users of an internet-based self-help intervention for COVID-19-related psychological distress. With regard to depressive symptoms, being younger predicted lower depressive symptoms after the 3-week intervention. With regard to resilience, higher motivation to use the intervention and better emotion regulation skills pre-treatment predicted higher resilience after the 3-week intervention.

We found that higher age was associated with worse treatment outcomes regarding depressive symptoms. This finding is inconsistent with previous research on predictors of internet-based self-help interventions for depression, in which age was not predictive of treatment outcome (63–67). The present finding is not straightforward to explain but could be related to the specific circumstances of the COVID-19 pandemic. A possible explanation could be a differential influence of various COVID-19-related stressors on psychological distress depending on age and that the intervention under study provided better support in dealing with certain stressors. For example, in a sample of 22-year-olds, secondary consequences of the COVID-19 pandemic, such as disruption of lifestyle or economic disruption were more strongly associated with psychological distress than COVID-19-related health risk exposures (68). Moreover, in one study, avoidant coping moderated the relationship between COVID-19 related psychological distress and depressive symptoms more strongly in younger adults compared to older adults (69). Therefore, in an uncontrolled setting, younger adults might benefit more from an intervention fostering adaptive coping than older adults. Given that research increasingly suggests that young adults are particularly affected mentally by the COVID-19 pandemic, the present finding is promising, despite the difficult explanation (29–32). However, since the ROCO intervention did not significantly reduce depressive symptoms and the present study was uncontrolled, such improvements could also be observed in younger adults not using the intervention.

Regarding resilience, we found that higher motivation to use the intervention and better emotion regulation skills pre-treatment predicted better treatment outcome. To date, there have been no studies examining predictors of treatment outcome in interventions promoting resilience, let alone internet-based interventions. However, in an internet-based self-help intervention for stress, higher motivation seemed to predict better adherence (70). Accordingly, it could be assumed that the effect of higher motivation on treatment outcome regarding resilience is mediated by adherence in our study as well. Yet, this assumption is not supported by our data, as the number of completed modules did not predict the treatment outcome in terms of resilience [*b*(SE) = 0.162 (0.226), *p* = 0.475]. However, these results could be attributed to the fact that we measured adherence only by the number of completed modules. Some studies point out that adherence involves much more than mere technological usage (71, 72). Therefore, it could be possible that highly motivated participants are otherwise more engaged with the internet-based intervention, for example, by addressing the content of the intervention in more depth or implementing it more thoroughly in their daily lives, which in turn could improve treatment outcome.

In addition to motivational conditions, better pre-treatment emotion regulation skills also appear to predict better treatment outcomes in an internet-based intervention for COVID-19 related psychological distress in terms of resilience. The better treatment outcome regarding resilience in participants with better pre-treatment emotion regulation skills could be caused by so-called capitalization. Capitalization describes the fact that pre-existing strengths of patients are reinforced and built on in therapy (73). In one study, tailoring treatment by focusing on patients' respective strengths rather than on their respective deficits led to better treatment outcomes in depressed patients (74). Since the intervention under study focuses, among other aspects, on building emotion regulation skills, it could be argued that emotion regulation skills were capitalized in participants with better pre-treatment emotion regulation skills. Previous research found that emotion regulation skills are associated with higher resilience (75) and better emotional adjustment (76). Therefore, capitalizing emotion regulation skills might lead to benefits in resilience. In conclusion, it appears that in the present study, participants with higher pre-treatment resources (motivation or emotion regulation skills) improved more during an internet-based self-help intervention regarding resilience in an uncontrolled setting.

In the current study, multiple possible predictor variables did not predict post-treatment depressive symptoms and resilience. For example, female gender predicted neither treatment outcome. This finding is consistent with studies that found no effect of female gender on treatment outcome (63–65, 77). However, there are also some studies that have shown that female gender predicted better treatment outcome (78–80).

The current study comes with several limitations. First, our sample was relatively small for predictor analysis. Our analyses might have been underpowered since predictor effects in internet-based interventions tend to be small. In addition, the small sample size in combination with the applied prediction procedure leads to an increased chance of incidental findings. Moreover, as only participants with complete data sets were included in the analysis, sample size was further reduced for some outcomes due to drop-out. To prevent further reduction of our sample, other methods (e.g., multiple imputation) could have been used to address missing data instead of listwise deletion. However, we decided to do a completer analysis instead of an intention-to-treat analysis. Second, participants in the delayed treatment group [*M (SD)* = 2.89 (2.53)] completed significantly less modules than participants in the immediate treatment group *[M (SD)* = 4.15 (2.27); *t*_(104.1)_ = 2.719, *p* = 0.009], whereas the effect size was *d* = 0.52 (medium effect size). One possible reason for this result could be that the burden of the participants in the delayed treatment group has already decreased during the waiting period. Accordingly, participants in the immediate treatment group showed higher mean depression scores [*M* (*SD*) = 11.13 (4.36)] than participants in the delayed treatment group [*M* (*SD*) = 9.60 (3.89)]. However, this difference was not significant (*t*_(102.1)_ = 1.908, *p* = 0.055).The current sample might have been already less burdened at pre-treatment, and therefore might not be representative of people with COVID-19 related psychological distress actively seeking support. Third, we relied only on self-report outcome measures and did not conduct a clinical assessment. Accordingly, responses could be subjectively biased. This could particularly concern information on psychological burden. Fourth, despite several analyses, we did not correct for multiple tests. Accordingly, this could result in type 1 errors. Nonetheless, since analyses were exploratory, avoiding type 2 errors is crucial. However, results must therefore be considered as hypotheses generating and not as definitive findings (81). Fifth, we have not analyzed moderators of treatment outcome. Therefore, we cannot answer whether the ROCO intervention was more effective for certain participants (e.g., younger adults) when compared to a control group. Based on our analyses we can only draw preliminary conclusions about which participants benefitted more in an uncontrolled setting. Sixth, we refrained from using modern prediction methods which might lead to difficulties in replicating our results (56, 82).

## Conclusion

Despite the limitations mentioned above, the current study gives preliminary evidence on the relationship between participant characteristics and treatment outcome in internet-based self-help interventions for COVID-19 related distress. One promising finding is that young adults, who can be considered a psychologically vulnerable group in the COVID-19 pandemic, seem to improve more using such an intervention in terms of depressive symptoms. Moreover, participants with higher motivation and better pre-treatment emotion regulation skills seemed to be able to build on their strengths and showed better treatment outcome in terms of resilience. Therefore, it could be beneficial to tailor interventions to respective strengths of the participants in order to promote resilience. However, further studies are needed to make informed decisions about the relationship of participant characteristics and treatment outcome in internet-based self-help interventions for COVID-19 related psychological distress. First, in further studies, the hypotheses generated in this exploratory analysis should be verified. Second, further studies should be conducted in a controlled setting to draw conclusions about the individuals for whom such an intervention is most effective.

## Data Availability Statement

The raw data supporting the conclusions of this article will be made available by the authors, without undue reservation.

## Ethics Statement

The studies involving human participants were reviewed and approved by Ethics Committee of the Canton of Bern. The patients/participants provided their written informed consent to participate in this study.

## Author Contributions

NB drafted the manuscript and was responsible for its conceptualization, methodology, formal analysis, data curation, and writing. JH was responsible for data curation and writing—reviewing and editing. TB and HZ were responsible for project administration, resources, and writing—reviewing and editing. All authors contributed to the article and approved the submitted version.

## Funding

This work was entirely funded by the University of Bern. Open access funding was provided by the University of Bern.

## Conflict of Interest

The authors declare that the research was conducted in the absence of any commercial or financial relationships that could be construed as a potential conflict of interest. The reviewer TL declared a past collaboration with one of the authors TB to the handling editor.

## Publisher's Note

All claims expressed in this article are solely those of the authors and do not necessarily represent those of their affiliated organizations, or those of the publisher, the editors and the reviewers. Any product that may be evaluated in this article, or claim that may be made by its manufacturer, is not guaranteed or endorsed by the publisher.
