# Erratum: Age, motivation, and emotion regulation skills predict treatment outcome in an internet-based self-help intervention for COVID-19 related psychological distress

**DOI:** 10.3389/fpubh.2023.1182957

**Published:** 2023-03-20

**Authors:** Frontiers Production Office

**Affiliations:** Frontiers Media SA, Lausanne, Switzerland

**Keywords:** COVID-19, internet-based self-help, depressive symptoms (DSs), psychological distress, resilience

An omission to the funding section of the original article was made in error. The following sentence has been added: “Open access funding was provided by the University of Bern.”

The original article has been updated.

